# Lack of Ubiquitin Specific Protease 8 (USP8) Mutations in Canine Corticotroph Pituitary Adenomas

**DOI:** 10.1371/journal.pone.0169009

**Published:** 2016-12-22

**Authors:** Silviu Sbiera, Marianna A. Tryfonidou, Isabel Weigand, Guy C. M. Grinwis, Bart Broeckx, Sabine Herterich, Bruno Allolio, Timo Deutschbein, Martin Fassnacht, Björn P. Meij

**Affiliations:** 1 Department of Internal Medicine I, Division of Endocrinology and Diabetes, University Hospital Würzburg, University of Würzburg, Würzburg, Germany; 2 Department of Clinical Sciences of Companion Animals, Faculty of Veterinary Medicine, Utrecht University, Utrecht, The Netherlands; 3 Departement of Pathobiology, Veterinary Pathology Diagnostic Center, Faculty of Veterinary Medicine, Utrecht University, Utrecht, The Netherlands; 4 Laboratory for Pharmaceutical Biotechnology, Faculty of Pharmaceutical Sciences, Ghent University, Ghent, Belgium; 5 Clinical Chemistry and Laboratory Medicine, University Hospital Würzburg, University of Würzburg, Würzburg, Germany; 6 Comprehensive Cancer Center Mainfranken, University of Würzburg, Würzburg, Germany; Colorado State University, UNITED STATES

## Abstract

**Purpose:**

Cushing’s disease (CD), also known as pituitary-dependent hyperadrenocorticism, is caused by adrenocorticotropic hormone (ACTH)-secreting pituitary tumours. Affected humans and dogs have similar clinical manifestations, however, the incidence of the canine disease is thousand-fold higher. This makes the dog an obvious model for studying the pathogenesis of pituitary-dependent hyperadrenocorticism. Despite certain similarities identified at the molecular level, the question still remains whether the two species have a shared oncogenetic background. Recently, hotspot recurrent mutations in the gene encoding for ubiquitin specific protease 8 (USP8) have been identified as the main driver behind the formation of ACTH-secreting pituitary adenomas in humans. In this study, we aimed to verify whether USP8 mutations also play a role in the development of such tumours in dogs.

**Methods:**

Presence of USP8 mutations was analysed by Sanger and PCR-cloning sequencing in 38 canine ACTH-secreting adenomas. Furthermore, the role of USP8 and EGFR protein expression was assessed by immunohistochemistry in a subset of 25 adenomas.

**Results:**

None of the analysed canine ACTH-secreting adenomas presented mutations in the USP8 gene. In a subset of these adenomas, however, we observed an increased nuclear expression of USP8, a phenotype characteristic for the USP8 mutated human tumours, that correlated with smaller tumour size but elevated ACTH production in those tumours.

**Conclusions:**

Canine ACTH-secreting pituitary adenomas lack mutations in the USP8 gene suggesting a different genetic background of pituitary tumourigenesis in dogs. However, elevated nuclear USP8 protein expression in a subset of tumours was associated with a similar phenotype as in their human counterparts, indicating a possible end-point convergence of the different genetic backgrounds in the two species. In order to establish the dog as a useful animal model for the study of CD, further comprehensive studies are needed.

## Introduction

Cushing’s disease (CD) in humans is a rare disease, with an annual incidence of 2–4 cases/million [1, 2]. It is characterized by an autonomous secretion of adrenocorticotropic hormone (ACTH) by a pituitary adenoma, resulting in an adrenal-derived glucocorticoid excess. While the clinical and histological phenotype is similar, canine CD (also frequently called in veterinary medicine pituitary-dependent hyperadrenocorticism) has an estimated incidence of 1000–2000 cases/million is therefore a much more common disorder [3]. In both species, overproduction of ACTH and cortisol leads to similar clinical manifestations including abdominal obesity, hypertension, muscle atrophy, and an increase in patient overall mortality [3]. The therapeutic approach to corticotroph adenomas in humans and dogs differ for several reasons among which are availability, sensitivity, and costs of diagnostic procedures (e.g. imaging) and therapeutic interventions (surgical or medical). In humans, the treatment of choice is selective transsphenoidal adenomectomy, resulting in high initial remission rates [4]. Patients with inoperable tumours or recurrent disease are candidates for focused radiotherapy and/or medical therapy. The latter may ameliorate the clinical symptoms through inhibition of pituitary ACTH release (i.e., dopamine agonists, somatostatin analogues), glucocorticoid receptor (GR) action (i.e., mifepristone), or adrenal cortisol synthesis (i.e., metyrapone, etomidate, mitotane) [5, 6]. In dogs, the main treatment usually consists of medical therapy with drugs such as mitotane or trilostane [7] while to date hypophysectomy or radiotherapy are performed in a few specialized centres only [8, 9].

The relatively high incidence of canine pituitary-dependent hyperadrenocorticism makes the dog an obvious model for pathogenetic studies. However, there are some differences between dogs and humans that may hamper direct extrapolation of findings from one species to another. These differences are partly related to differences in the distribution of cells in the pituitary gland. The pituitary gland consists of an anterior, glandular lobe (consisting of endocrine cells secreting six different trophic hormones including ACTH) and a posterior, neuronal lobe (secreting oxytocin and vasopressin) [10]. Inbetween these two lobes resides the intermediate zone (*pars intermedia*). While in humans only α-melanocyte-stimulating hormone (α-MSH) is secreted in the latter part, the canine *pars intermedia* consists of two groups of cells: the predominant A-cells (secreting α-MSH as in humans), and to a lesser extent the B-cells (secreting ACTH) [11]. The ACTH-secreting cells of the anterior lobe react to the stimulatory effect of the hypothalamic corticotropin-releasing hormone (CRH), whereas the *pars intermedia* cells are inhibited by dopamine that is secreted from the arcuate nucleus [12]. In humans, the corticotroph adenomas originate from the anterior lobe, whereas in dogs they can develop from either anterior (90%) or intermediate zone cells (10%). Several investigators have attempted to distinguish adenomas originating from one of these lobes, but until now a reliable distinction cannot be made. However, since *pars intermedia* adenomas only account for 10% of canine corticotroph adenomas, the dog remains an interesting model for providing mechanistic data, hopefully also enabling a better understanding of human CD.

**Fig 1:**
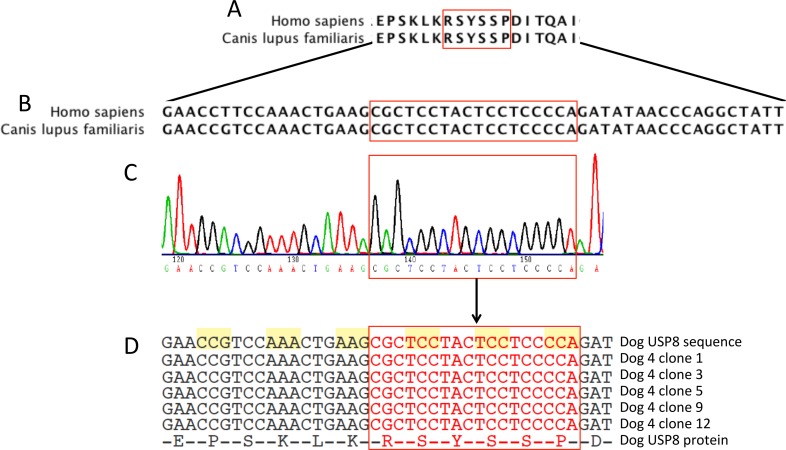
Sanger sequencing results of the 14-3-3 binding site of canine ubiquitin specific protease 8 (USP8). Representation of protein (A) and DNA (B) USP8 sequence consensus around the 14-3-3 binding motif (red rectangle) between *Homo sapiens* (region corresponding to the amino-acids 715–720) and *Canis lupus familiaris* (region corresponding to the amino-acids 713–718). Sanger sequencing of directly (C) and cloning PCR (D) amplified DNA showed no mutations present in the 14-3-3 binding motif (red rectangle).

Recently, our group and others [13–16] have identified recurrent mutations in the 14-3-3 binding site of the ubiquitin specific protease 8 (USP8) gene as the main driver behind the formation of corticotroph adenomas in humans. These mutations seem to affect the recycling speed of the epidermal growth factor receptor (EGFR) to the membrane of the ACTH-secreting pituitary adenoma cells, thus prolonging its effects [13, 14]. As a connection of EGFR with the pathogenesis of corticotroph adenomas has already been reported in both humans and dogs [17] we hypothesized that the underlying mechanism for adenoma development might be similar in both species. Of note, the 14-3-3 binding site of USP8 is highly conserved between the two species both at gene and protein level (Fig 1A and 1B). Accordingly, we analysed the presence of USP8 hot-spot mutations in canine ACTH-secreting adenomas to answer the question whether USP8 mutations may also play a role in the development of corticotroph adenomas in dogs. Furthermore, we studied the role of USP8 and EGFR protein expression independent of USP8 mutation status in corticotroph adenomas.

## Material and Methods

### Animals

Pituitary adenoma tissue was collected from client-owned dogs (n = 38, that were treated at the Department of Clinical Sciences of Companion Animals, Faculty of Veterinary Medicine, Utrecht University according to previously published protocols [18, 19]. Thirty-five of these dogs underwent transsphenoidal hypophysectomy as treatment for CD. In three dogs with biochemically confirmed CD, pituitary adenoma tissue was collected with the owner’s consent immediately (within 30 minutes) after euthanasia due to terminal disease. Clinical characteristics of the dogs are depicted in Table 1. The diagnosis of hypercortisolism was based on an elevated urinary corticoid-to-creatinine ratio (UCCR; ≥10×10^−6^), combined with non-suppressed ACTH and non-suppressed cortisol after high-dose dexamethasone (0.1 mg/kg) injection [20]. The pituitary origin of the disease was further supported by ultrasonographic visualization of the adrenals and pituitary imaging as previously described [21–23]. In dogs suffering from CD, enlarged pituitaries were distinguished from non-enlarged pituitaries by their pituitary height/brain area (P/B) value [24].

**Table 1:** Clinical characteristics of the patients included in the study.

No	Breed	Age	Sex	BW	UCCR^a^	Dex ST^b^	Pituitary size^c^	P/B^d^
		(yr)		(kg)	(X 10^−6^)	(%)	(h x w x l; mm)	(mm^-1^)
1	Belgian shepherd dog	11.9	MC	39	23	70.9	8.2 x 9.7 x 10	0.49
2	English cocker spaniel	5.0	F	17.6	112.5	96.7	4.4 x 6.5 x 5	0.31
3	Softcoated wheat terrier	8	F	14.5	44	72.7	7.8 x 8.8 x 6	0.51
4	Mixed breed	12.2	M	15.3	64	95.6	6.7 x 7.0 x 7.0	0.39
5	Pitbull terrier	8.4	MC	23	244	90.6	11 x 13.5 x 11	0.64
6	Irish Setter	9.4	F	30	26.5	83.8	5.7 x 7.1 x 6.5	0.33
7*	Beagle	14.5	FC	11.2	50	38	8.5 x 8.8 x 9.2	0.5
8	Am. Staffordshire terrier	9.2	MC	40.8	1.7	NA	8.5 x 17 x 13.1	0.47
9	Whippet	10.7	M	14.8	21.9	79.0	11.5 x 8.6 x 8.8	0.64
10	Chesapeake Bay retriever	7.1	M	41.5	65	66.2	11.9 x 13.9 x 16.5	0.70
11	French Bulldog	5.6	FC	11.6	134.8	65.2	7.9 x 7.6 x 8.4	0.44
12	Bordeaux dog	8.5	M	53.6	23.5	87.7	8.0 x 11.0 x 8.8	0.36
13*	French Bulldog	7.2	M	16.3	128.4	30.5	16.8 x 11.1 x 12	1.04
14	Mixed breed	9.1	FC	18.3	53.5	51.4	6.4 x 6.7 x 7.6	0.41
15*	Mixed breed	9.0	M	31.4	11.5	86.0	20.5 x 17.7 x 21.0	1.14
16	Jack Russell terrier	8.5	FC	11.4	NA	NA	6.1 x 9.6 x 8.1	0.39
17	Labrador retriever	8.4	F	43.2	175.0	82.3	9.5 x 10.5 x 11.2	0.50
18	Mixed breed	6.9	M	32.9	133.9	89.7	7.5 x 9.1 x 8.0	0.45
19	Mixed breed	5.0	FC	29.8	125.5	2.8	12.0 x 14.0 x 14.0	0.62
20	English Cocker Spaniel	5.8	F	16.7	96.1	69.4	14.2 x 17.4 x 17.0	1.10
21	Pinscher	10.6	F	9.0	2799.4	85.9	5.9 x 5.9 x 7.0	0.49
22	Jack Russell terrier	11.7	MC	13.0	13.5	74.1	12.6 x 13.4 x 16.2	0.74
23	Maltese dog	10.4	FC	3.7	213.5	67.2	13.8 x 14.4 x 15.3	0.91
24	Jack Russell terrier	10.9	FC	8.5	26.0	88.1	6.3 x 7.1 x 8.7	0.38
25	Beagle	12.1	M	24.0	NA	NA	22.5 x 21.3 x 20.7	1.25
26	Vizsla	14.0	MC	21.0	NA	NA	15.0 x 21.4 x 18.3	0.98
27	German Pointer	10.8	FC	31.0	22.1	99.1	5.8 x 8.0 x 7.0	0.32
28	Bernese Mountain dog	6.7	F	48.0	100.0	61.0	11.7 x 13 x 11.7	0.69
29	Labrador retriever	9.4	M	34.5	19.7	81.7	7.1 x 9.2 x 9.5	0.44
30	Mixed breed	10.4	MC	5.4	62.5	69.6	5.3 x 5.8 x 6.0	0.38
31	Petit Basset Griffon Vend.	7.8	MC	13.9	302.5	33.2	5.0 x 6.1 x 4.7	0.30
32	Mixed breed	11.9	FC	11.5	26.0	NA	7.1 x 7.6 x 7.1	0.46
33	Stabyhoun	10.5	FC	23.4	34.5	89.0	5.4 x 7.6 x 6.2	0.35
34	Labrador retriever	8.3	FC	36.5	30.7	88.3	11.5 x 11.8 x 11.6	0.56
35	Beagle	8.2	M	23.2	74.5	75.8	16.6 x 14.6 x 16.0	1.00
36	Miniature Pinscher	3.7	MC	6.7	18.6	NA	5.0 x 6.2 x 8.0	0.37
37	Maltese dog	7.0	M	6.0	330.4	8.3	9.9 x 8.3 x 9.3	0.75
38	Mixed breed	6.5	M	12.8	81.0	60.5	14.9 x 13.8 x 16.1	0.99

Abbreviations: C, castrated; F, female; M, male; NA, not available

^a^UCCR, urinary corticoid-to-creatinine ratio (reference<10×10^−6^); values are the mean of 2 morning urine samples with a 1-d interval.

^b^High-dose dexamethasone (DEX) suppression test (ST), % suppression of UCCR after dexamethasone.

^c^Pituitary sizes (h, height; w, width; l, length) in mm, as measured on computed tomography or magnetic resonance imaging

^d^Pituitary height/brain area value (x10^2^ mm^−1^), P/B ≤ 0.31 indicates a non-enlarged pituitary; P/B > 0.31 indicates an enlarged pituitary.

*Indicates 3 dogs with confirmed pituitary-dependent hyperadrenocorticism that were not hypophysectomized but euthanized, with collection of pituitary tissue immediately post-mortem.

### Pituitary tissue specimens

Immediately after collection, specimens of pituitary adenoma tissue were fixed in 4% neutral buffered formaldehyde, embedded in paraffin, and consecutive sections were used for histology and immunohistochemistry. Representative adenoma tissue samples were snap-frozen and stored in liquid nitrogen until subsequent analysis. A bio-bank of pituitary adenoma samples was used to generate two study cohorts. In a first round 18 samples were subjected to sequencing of the USP8 hotspot of which 14 also underwent immunohistochemical analysis of USP8 and EGFR expression. In a second round, 20 additional ACTH-secreting pituitary adenomas were subjected to sequencing of the USP8 hotspot to confirm the results of the first round and further 12 samples where FFPE tissues were available underwent immunohistochemical analysis of USP8 and EGFR expression.

As control tissue, anterior lobes of normal pituitary glands were obtained from 7 healthy dogs (4 male and 3 female dogs; 1 Bouvier, 5 Labrador retrievers, and 1 Beagle; median age 33 months, range 8 to 44 months) euthanized in other circumstances, unrelated to hyperadrenocorticism. The study protocol was approved by the Ethics Committee on Animal Experimentation of Utrecht University, the Netherlands, in accordance to the 3R-policy (DEC-number 2007.III.06.080).

### USP8 sequencing

DNA was extracted from fresh-frozen dog adenoma tissue using a salt extraction method [25]. A fragment of 382bp around the 14-3-3 binding site of canine USP8 was then amplified using the primer pair: USP8_F (exonic: chr.30:16,399,619–16,399,638): TTCTTGACCCAATCACTGGA and USP8_R (intronic: chr.30:16,399,979–16,400,000): GGCTGGTATAACCATCCTCAGA. PCR was performed on 20 ng of DNA from each sample in a final volume of 25 μl containing 1.5 mM MgCl_2_, 0.2 μM of each primer, 200 μM dNTPs, and 1 U Taq DNA Polymerase. Cycling conditions were: 35 cycles, denaturing at 93°C (30 sec), annealing at 58°C (30 sec), and elongation at 72°C (30 sec). Direct sequencing of the PCR products was performed using the QuickStart Cycle Sequencing Kit (ABSciex) on a CEQ8000 DNA Analyzer (ABSciex). Primer USP8_F was used for sequencing. To exclude possible variant alleles present in a low proportion of neoplastic cells, colony PCR sequencing was performed exemplary for the DNA of two of the canine corticotroph adenomas (dog 4 and 5). In short, the 14-3-3 binding site of canine USP8 was amplified by PCR using the “high fidelity” Q5 High-Fidelity DNA polymerase (New England Biolabs). For this, the same primers and conditions as mentioned above were applied. The PCR products were then cloned using the TOPO TA Cloning Kit for Sequencing (Life Technologies) according to the company instructions. Fourteen clones for dog 4 and 13 clones for dog 5 were analysed by direct sequencing using the QuickStart Cycle Sequencing Kit on the CEQ8000 DNA Analyzer. Primer USP8_F was used for sequencing.

To evaluate the presence of (non) synonymous mutations in the USP8 gene outside the 14-3-3 binding site, targeted sequencing of the entire CDS of the USP8 gene was performed in ten ACTH-secreting adenomas (samples 1, 2, 4, 5, 6, 8, 10, 13, 14 and 16) as described previously [26]. A minimum sequencing depth of 10x was reached for nearly the entire CDS region (median: 92.7%, range: 79.0% - 96.9%). Variants were called and filtered as described previously [27].

### Histology and immunohistochemistry

The diagnosis of pituitary adenoma was made on haematoxylin and eosin (H&E) stained tissue sections by a board-certified veterinary pathologist (GG). The diagnosis of a corticotroph adenoma was confirmed at the time of diagnosis by immunostaining for ACTH, α-MSH, and growth hormone (GH) as previously described [28]. For our study-related correlation purposes, the qualitative evaluation of ACTH and α-MSH was transformed in semi-quantitative values with “negative” = 0, “mild positive” = 1, “positive” = 2 and “strongly positive” = 3. Half scores were allocated if the original evaluation was not categorical (e.g. “positive to strongly positive” = 2.5).

Pituitary tissue of healthy dogs was also H&E stained and histology confirmed normal pituitary glands.

**Fig 2:**
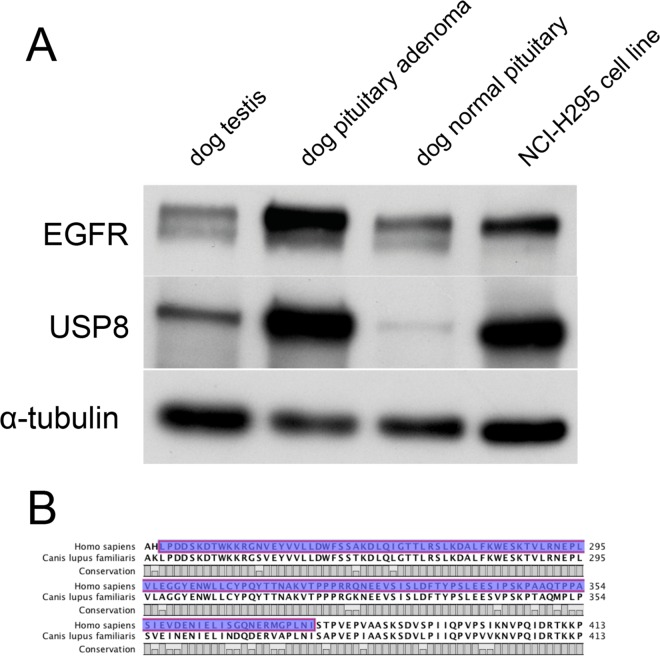
Interspecies specificity of the antibodies used for immunohistochemistry. (A) Western blot analysis of EGFR, USP8 and α-tubulin expression proteins extracted from dog testis, dog pituitary adenoma and normal pituitary gland and from the human adrenocortical cell-line NCI-H295. (B) Protein sequence conservation status between human (upper sequence) and dog (lower sequence) in the area of the anti human USP8 antibody epitope (amino-acids 239–377, blue colour).

The antibodies used in immunohistochemistry were tested beforehand for inter-species specificity in immunoblotting, using proteins extracted from dog testis, dog pituitary adenoma and normal pituitary gland and from the human adrenocortical cell-line NCI-H295 (Fig 2). Immunohistochemistry for USP8 and EGFR was performed on 26 (out of 38, where FFPE tumour material was still available) dog corticotroph adenomas and seven normal dog pituitary glands, applying rabbit polyclonal antibodies against USP8 (Sigma, HPA004869) and EGFR (Santa-Cruz, sc-03) both diluted 1:500 in PBS. Subsequent detection was performed using the EnVision + System Anti-rabbit (Dako, Glosdrup, Denmark) and nuclei counter-stained with haematoxylin as described [29]. Each of these antibody concentrations was determined in preliminary staining experiments, using serial dilutions on normal canine testes (for USP8) and human adrenocortical carcinoma (for EGFR). The N-Universal Negative Control Rabbit antibody (Dako) served as negative control. Cytoplasmic and nuclear staining in the USP8-stained sections and cytoplasmic, membranous and nuclear staining in the EGFR-stained sections, were evaluated semi-quantitatively, after four separate evaluation rounds, by an experienced, board-certified veterinary pathologist (G.G.). Relative cytoplasmic and nuclear immunoreactivity was graded from 3 when revealing the most prominent staining within the group of adenomas to 0 when staining was absent (Table 2). Regions with adenoma cells were identified and distinguished from areas with unaffected pituitary cells based on evaluation of H&E staining of a consecutive slide of each case.

**Table 2:** Histopathology and immunohistochemistry of pituitary tissue specimens.

No	Histological	Immunohistochemistry							
	diagnosis (H&E)	ACTH score	α-MSH score	GH	EGFRmem.	EGFRcyto.	EGFR nuc.	USP8cyto.	USP8nuc.
1	Adenoma with cysts	2	2	-	2	2	1	2	1
2	Adenoma	2	2	-	3	3	1	3	2
3	NA	NA	NA	NA	NA	NA	NA	NA	NA
4	Adenoma	1	1	-	1	1	1	2	1
5	Adenoma	2	2	-	2	2	1	3	2
6	Adenoma	1	1	-	2	2	1	3	2
7	NA	NA	NA	NA	NA	NA	NA	NA	NA
8	Adenoma	3	1	-	1	1	1	1	2
9	Adenoma	2.5	2.5	-	2	2	1	1	3
10	Adenoma	2.5	3	-	NA	NA	NA	NA	NA
11	Adenoma	2.5	2.5	-	3	2	1	3	2
12	Adenoma with cysts	3	2	-	3	3	1	2	2
13	Infiltrative adenoma	1	2.5	-	3	2	0	2	1
14	Adenoma	2.5	2.5	-	NA	NA	NA	NA	NA
15	Adenoma with malignant characteristics	0.5	0.5	-	1	1	0	2	0
16	Adenoma	3	1	-	2	1	0	2	2
17	Adenoma	3	2.5	-	3	3	0	2	2
18	Adenoma	2	3	-	NA	NA	NA	NA	NA
19	Adenoma	3	2	-	1	1	0	1	0
20	Adenoma with malignant characteristics	0.5	0.5	-	3	2	0	1	1
21	Adenoma	2	2	-	3	2	0	1	1
22	Adenoma	2	NA	-	NA	NA	NA	NA	NA
23	Adenoma	3	NA	-	NA	NA	NA	NA	NA
24	Adenoma	3	3	-	3	2	0	1	1
25	Adenoma	2	2	-	2	1	0	1	1
26	Adenoma	1	1	-	NA	NA	NA	NA	NA
27	Adenoma	3	2	-	2	1	0	1	3
28	Adenoma with malignant characteristics	3	2.5	-	3	2	0	2	2
29	Adenoma	2	2	-	2	1	0	1	3
30	Adenoma	3	2	-	NA	NA	NA	NA	NA
31	Adenoma	2	0	-	NA	NA	NA	NA	NA
32	Adenoma	1	2	-	3	2	0	2	2
33	Adenoma	3	2	-	3	2	0	1	2
34	Adenoma	1	1	-	1	1	0	2	1
35	Adenoma	2	2	-	3	2	0	1	1
36	Adenoma	NA	NA	NA	NA	NA	NA	NA	NA
37	Adenoma	NA	NA	NA	NA	NA	NA	NA	NA
38	Adenoma	0	0	-	0	0	0	1	1

ACTH = adrenocorticotropic hormone; α-MSH = α-melanocyte stimulating hormone; GH = growth hormone

USP8 = ubiquitin specific protease 8; EGFR = epidermal growth factor receptor

mem. = membrane; cyto. = cytoplasm; nuc. = nucleus.

NA = not available

### Statistical analyses

Comparisons between two groups (adenoma versus normal pituitary) were performed using the non-parametric Mann-Whitney test. For correlation between different categorical variables the Spearman r correlation test was used. All statistical analyses were conducted using the GraphPad Prism 6.0 Software for Mac (Graphpad Inc, La Jolla, USA).

## Results

### USP8 sequencing

Sanger sequencing of the DNA obtained from the first cohort of 18 canine ACTH-secreting adenomas revealed no mutation in the 14-3-3 binding site of the USP8 gene (Fig 1C). This result was also confirmed in a second round, now sequencing 20 additional DNA samples from canine corticotroph adenomas. Exemplary sequencing of 27 PCR clones from two of the samples attested that not even a subgroup of neoplastic cells carried these mutations (Fig 1D). Additional next generation sequencing of the whole CDs of the USP8 gene in 10 exemplary dogs where enough genetic material was available showed absence of any mutation in the gene also outside the 14-3-3 binding site.

### Protein expression pattern in normal pituitary glands and corticotroph adenomas

The interspecies specificity of the EGFR antibody has been already demonstrated by fluorescent immunohistochemistry in a study by Fukuoka *et al* [17] and our immunoblotting experiment confirmed this (Fig 2A). We could also demonstrate that the USP8 antibody we have used in our previous study on human corticotroph tumours [13] was also specific for the canine protein (Fig 2A), unsurprisingly as its epitope recognizes a highly conserved protein sequence in both species (Fig 2B).

**Fig 3:**
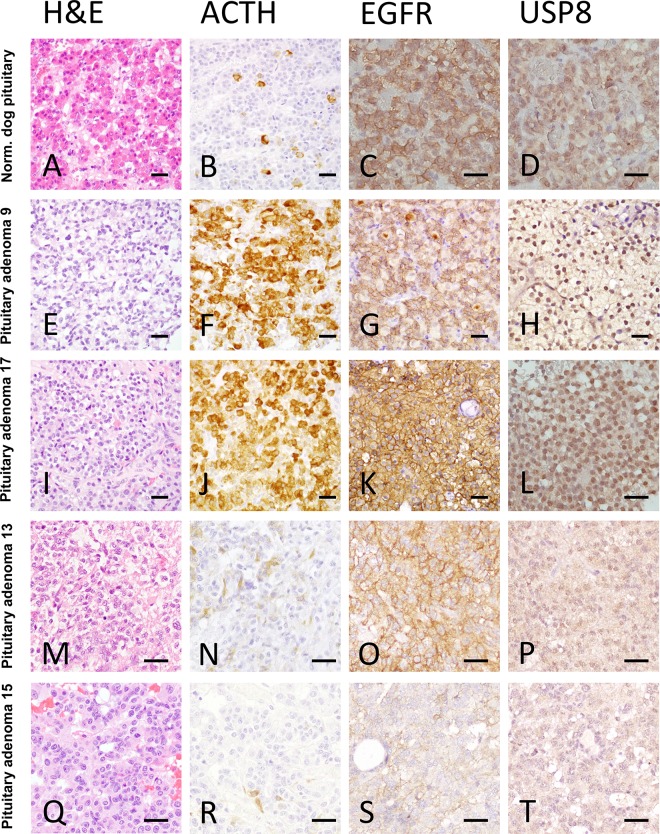
Immunohistological characterization of canine pituitary tissues. Normal canine pituitary gland (A-D) and canine ACTH-secreting pituitary adenomas (E-T) stained with Haematoxylin/Eosine (A, E, I, M and Q), ACTH (B, F, J, N and R), EGFR (C, G, K, O and S) and USP8 (D, H, L, P and T). The ACTH-secreting pituitary adenomas have been stratified according to their nuclear USP8 staining from H-score 3 (H) to H-score 0 (T). Scale bars = 20μm.

**Fig 4:**
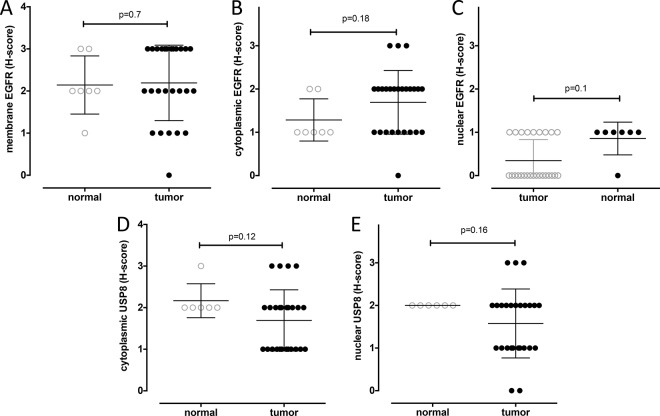
Staining intensity distribution in normal canine pituitary glands vs. canine ACTH-secreting pituitary adenomas. H-scores distribution of EGFR expression in the membrane (A), cytoplasm (B) and nucleus (C) and USP8 in the cytoplasm (D) and nucleus (E) of normal dog pituitary cells (empty circles) and ACTH producing pituitary adenoma cells (full circles).

The histology of anterior pituitary glands in the normal dogs revealed a specific pattern containing basophilic, acidophilic, and chromophobic cells (Fig 3A). In contrast, most of the corticotroph adenoma cells were acidophilic or basophilic, and the respective tumors showed a disrupted cellular pattern (Fig 3E, 3I, 3M and 3Q). In the normal pituitary, ACTH production was restricted to specialized cells (Fig 3B) whereas in the adenomas the neoplastic cells produced ACTH (Fig 3F, 3J, 3N and 3R; Table 2). Immunohistological staining for EGFR showed localization of this molecule in all subcellular compartments, and this was true for both normal and adenoma tissue (Fig 3C, 3G, 3K, 3O and 3S) showing similar expression levels for membranous (mean expression 2.14±0.69 vs 2.19±0.89, p = 0.7, respectively, Fig 4A), cytoplasmic (mean expression 1.28±0.48 vs 1.69±0.73, p = 0.18, respectively, Fig 4B) and nuclear (0.85±0.37 vs 0.34±0.48, p = 0.1, respectively, Fig 4C) localization. USP8 in both normal pituitary and adenoma was expressed both in the cytoplasm and the nuclei (Fig 3D, 3H, 3L, 3P and 3T; Table 2). There was no statistical difference between the score intensity in normal pituitary vs. adenoma tissue (mean cytoplasmic USP8 2.16±0.40 vs 1.69±0.73, p = 0.12, respectively, Fig 4D, and mean nuclear USP8 2.00±0.00 vs 1.57±0.80, p = 0.16, respectively, Fig 4E). However, while the USP8 staining was comparable in all normal pituitary samples, its expression profile (both cytoplasmic and nuclear) was highly variable in the ACTH-secreting adenomas compared to the normal gland.

### Correlation between protein expression and clinical-pathological data

**Fig 5:**
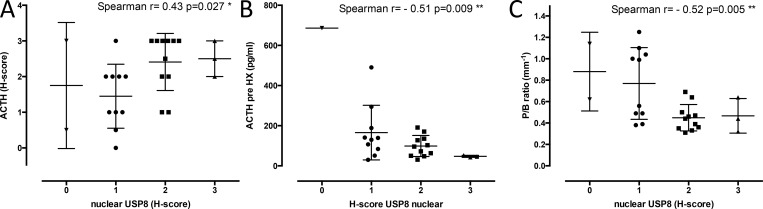
Influence of nuclear USP8 protein expression on the pathophysiology of canine ACTH-secreting pituitary adenomas. Correlations between nuclear USP8 staining intensity (X-axis) and ACTH staining intensity (A), blood ACTH levels pre-surgery (B) and tumour size reported as P/B ratio (C).

Analysis of EGFR and USP8 staining intensities and clinical-pathological data of the respective adenomas revealed several correlations. We found no correlation between any EGFR or cytoplasmic USP8 protein staining and clinical-pathological data. However, higher nuclear USP8 correlated directly with the intensity of ACTH staining of the tumours (Spearman r = 0.43, p = 0.027*, Fig 5A). Intriguingly, the nuclear USP8 intensity correlated inversely with the pre-surgery blood levels of ACTH in the same dogs (Spearman r = - 0.51, p = 0.009**, Fig 5B). The reason for this apparent discrepancy was the fact that increased nuclear USP8 was associated with a smaller size of the tumours (Spearman r = - 0.52, p = 0.005**, Fig 5C).

## Discussion

The thousand-fold higher incidence of CD in dogs compared to humans makes this species a obvious model to study the underlying pathogenesis of corticotroph adenomas[3]. In both species, the disease is similarly characterized through clinical manifestations [2] and, to some degree, common denominators that have been identified at the molecular level [17]. Nevertheless, while the phenotypic manifestation of the disease may be similar, the question arises whether the genetic aetiology is truly the same. One argument against a similar origin is the much higher frequency of CD in dogs than in humans, however, it is known that dogs generally have a higher predisposition to cancer development due to a limited genetic pool caused by pedigree breeding [30]. Another impediment in comparing the genetic background of CD in the two species is the fact that for a long time even the genetic cause of CD in humans was unknown [16]. Recently, our group and others have identified recurrent mutations in the 14-3-3 binding site of USP8 as the main driver behind the formation of corticotroph adenomas in humans [13–15]. This binding site is highly conserved between several species including the dog [13]. In this study, we therefore tried to address the question whether human and canine CD are caused by the same hotspot mutations in the USP8 gene. In 38 canine corticotroph pituitary adenomas studied, however, Sanger sequencing of the region around the 14-3-3 binding site of USP8 revealed no mutation. As the majority of these tumours have been excised through transsphenoidal surgery, there is certainly a risk for contamination of the extracted DNA with cells of the healthy part of the pituitary and blood cells, potentially masking the possible genetic modifications in the tumour fraction. Therefore, we performed additional exemplary PCR cloning sequencing in two of the samples, which confirmed the lack of USP8 mutations previously shown by the original Sanger sequencing. We also performed NGS sequencing of all USP8 CDs in ten samples with enough genetic material and were able to exclude also the possibility that mutations in other regions outside the 14-3-3 binding site of the USP8 gene may be involved in the canine pituitary-dependent hyperadrenocorticism. In humans, the hot-spot mutations were found to result in an increased USP8 protein expression, clustering to the nucleus [13], that correlated in some publications with an increase in EGFR expression in the tumour tissue [14], while other studies could not confirm this correlation [31]. Consequently, the question aroused whether other causes may lead to a comparable effect on canine protein expression, despite a lack of hotspot mutations. Immunohistochemistry staining of EGFR in the canine tumours revealed protein localisation at all subcellular levels (membrane, cytoplasm and nucleus) and there was no significant difference regarding the levels of expression between normal pituitaries and pituitary adenomas. Previously, using the same antibody, Fukuoka et al. reported exclusive nuclear localization of EGFR in corticotroph pituitary adenomas both in humans and dogs as compared to other types of cancer [17]. However, this exclusive localisation has not been reported since in any study that analysed EGFR expression in this type of tumours. For example, two recently published studies on this subject in humans [14, 31] both show mostly cytoplasmic and membraneous localization of EGFR in corticotroph adenomas despite analysing a large number of tumours. One explanation for this discrepancy would be the fact that Fukuoka et al used fluorescence IHC that has the disadvantage that it has a narrower dynamic range, which makes it difficult to visualize rare and high abundant targets on the same slide and is susceptible to photobleaching and exposure time variations and in FFPE material also to tissue autofluorescence. There was also no correlation between EGFR staining and USP8 staining or clinical-pathological data, suggesting that in dogs EGFR may play an independent role from USP8 in line with other publications [31] but also contrary to what has already been published for human CD [14].

Immunohistochemistry staining of USP8 revealed that USP8 was localised in both the cytoplasm and the nucleus of normal pituitary and adenoma tissues. Moreover, there was also no statistical difference regarding the average staining intensity. However, if the different tumour samples were compared, nuclear USP8 staining was more heterogeneous, and nuclear expression as well as ACTH production within these cells highly correlated. In the same time higher nuclear USP8 expression correlated with a smaller tumour size, which may be why it also correlated significantly with a decreased amount of ACTH levels in the blood pre-surgery. This phenotype is reminiscent of the phenotype of human corticotroph tumours carrying the USP8 mutations [14, 15], suggesting that USP8 may nevertheless play an important role in the regulation of ACTH synthesis.

In conclusion, while the human and canine corticotroph adenomas do not seem to share the same genetic background, our molecular analyses show that USP8 protein expression seems to play a role for the development of CD in both species, suggesting a possible end-point convergence. However, in order to establish the dog an adequate animal model in the study of this devastating disease, further extensive genetic analyses of canine corticotroph adenomas and their influence on specific pathways are still necessary.
